# The speed of FtsZ treadmilling is tightly regulated by membrane binding

**DOI:** 10.1038/s41598-020-67224-x

**Published:** 2020-06-26

**Authors:** Daniela A. García-Soriano, Tamara Heermann, Ana Raso, Germán Rivas, Petra Schwille

**Affiliations:** 1 Department of Cellular and Molecular Biophysics, Max Planck Institute for Biochemistry, Martinsried, Munich, Germany; 2 Graduate School for Quantitative Biosciences (QBM), Ludwig-Maximillians-University, Munich, Germany; 3 Centro de Investigaciones Biológicas, Consejo Superior de Investigaciones Científicas (CSIC), Madrid, Spain; 4 Department of Engineering, Aarhus University, Aarhus, Denmark

**Keywords:** Biochemistry, Cell biology, Molecular biology

## Abstract

As one of the key elements in bacterial cell division, the cytoskeletal protein FtsZ appears to be highly involved in circumferential treadmilling along the inner membrane, yielding circular vortices when transferred to flat membranes. However, it remains unclear how a membrane-targeted protein can produce these dynamics. Here, we dissect the roles of membrane binding, GTPase activity, and the unstructured C-terminal linker on the treadmilling of a chimera FtsZ protein through *in vitro* reconstitution of different FtsZ-YFP-mts variants on supported membranes. In summary, our results suggest substantial robustness of dynamic vortex formation, where only significant mutations, resulting in abolished membrane binding or compromised lateral interactions, are detrimental for the generation of treadmilling rings. In addition to GTPase activity, which directly affects treadmilling dynamics, we found a striking correlation of membrane binding with treadmilling speed as a result of changing the MTS on our chimera proteins. This discovery leads to the hypothesis that the *in vivo* existence of two alternative tether proteins for FtsZ could be a mechanism for controlling FtsZ treadmilling.

## Introduction

FtsZ is a prokaryotic tubulin homologue that polymerizes via GTPase activity. FtsZ polymers interact with the peripheral membrane protein FtsA and the integral membrane protein ZipA, and serve as a scaffold for the recruitment and coordination of the septal peptidoglycan synthesis machinery during bacterial cell division^1,2^. The protein consists of two globular subdomains, N-and C-terminal, that can fold independently. The N-terminal subdomain comprises a GTP-binding and hydrolysis site, as well as residues enabling lateral self-interaction^3^. The C-terminal subdomain contains three essential regions for FtsZ self-assembly and interaction with division-associated proteins such as MinC, FtsA and ZipA. These are (i) an unstructured C-terminal linker (CTL) that supposedly weakens lateral interactions by electrostatic repulsion, modulating the on and off rates of monomers, as well as fragmentation and annealing processes;^4,5^ (ii) a C-terminal constant region (CTC); and (iii) a C-terminal variable region (CTV), both involved in regulating interactions with further cytoplasmatic or membrane proteins^6^.

Similar to eukaryotic cytoskeletal filaments, FtsZ polymerizes in a GTP- and monovalent cation-dependent manner, thereby assembling into short protofilaments^3,7,8^. When assembled into the bacterial Z-ring, these filaments turn over with a steady-state half-time of approximately 8 to 9 s^9,10^. Intriguingly, this progressive movement of FtsZ polymers appears to occur through a treadmilling mechanism, in which one end of the polymer grows (polymerization, plus end) and the other shrinks (depolymerization, minus end)^11^. Although both *in vivo*^12,13^ and *in vitro*^14,15^ data support this mechanism, knowledge is scarce about the factors modulating the dynamic properties of this system^12,14,15^.

To better understand the treadmilling dynamics of FtsZ in a quantitative manner, we investigated and dissected the influence of three factors on vortex dynamics: (i) membrane binding, (ii) GTPase activity, and (i) the unstructured C-terminal linker supposedly affecting lateral interactions. For this purpose, we employed a minimalistic *in vitro* reconstitution approach to examine these factors through protein domain-specific sequence alterations of a membrane-targeted FtsZ-YFP-mts (WT) chimera. We were able to show that membrane anchoring and dynamic lateral interactions are crucial for FtsZ ring formation. Furthermore, our results emphasize that both, the GTPase activity of FtsZ and the membrane binding capacity of the fused anchor directly and significantly influence the treadmilling speed, complementing *in vivo* published data^12^. In comparison with our previous study where we showed that FtsZ can intrinsically form FtsZ vortices^15^, this work provides a selective analysis of the main factors influencing and enabling treadmilling dynamics of FtsZ vortices.

## Results

### Deficient membrane binding and absence of the C-terminal linker impede FtsZ-ring formation

In recent years, two factors have been proposed to influence FtsZ assembly into dynamic vortices: membrane anchoring and lateral interactions^16^. However, so far, it remained somewhat ambiguous how exactly these factors contribute to the assembly and dynamics of the vortices. To dissect their respective roles for the formation of FtsZ *E. coli* rings, we generated two FtsZ-YFP-mts mutants with either a defective membrane-binding motif (FtsZ-YFP- mts^*[L629E]^) or an absent C-terminal linker known to regulate lateral interactions (FtsZ^Δ-Cterm^-YFP-mts).

**Figure 1:**
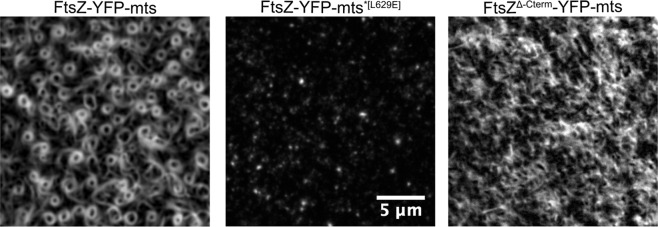
FtsZ-YFP-mts assembly into treadmilling rings is impaired when either membrane binding or the C-terminal linker is disrupted. Representative snapshots of the *in vitro* reconstitution of 0.5 µM FtsZ-YFP-mts (left panel), 0.5 µM FtsZ-YFP-mts^*[L629E]^ (middle panel), and 1 µM FtsZ^Δ-Cterm^-YFP-mts (right panel) on *E. coli* polar lipid extract membranes (4 mM GTP and 5 mM Mg^2+^). (Middle panel) Rather than self-organizing into dynamic FtsZ vortices, FtsZ-YFP-mts^*[L629E]^ binds as isolated patches to the *E. coli* polar lipid extract membrane. (Right panel) FtsZ^Δ-Cterm^-YFP-mts forms static filamentous structures on the supported lipid bilayer. These results thus suggest that effective membrane binding and dynamic self-assembly^26^ play a crucial role in the formation of FtsZ rings.

As illustrated in Fig. 1, both FtsZ-YFP-mts mutants displayed distinct and sequence-specific phenotypes when compared to the wild type protein (Fig. 1). In the case of FtsZ-YFP- mts^*[L629E]^, which possess a previously reported disruptive mutation in the membrane target sequence (mts) of the *E. coli* MinD^17^, only small protein patches appeared on the membrane surface (Fig. 1, middle panel). At the same time, sedimentation analysis showed that the polymerization properties of this mutant variant were significantly impaired when compared to the chimera with the native MinD mts (S1 Fig). Taken together, these results suggest that the inability of self-assembling into curved filaments or vortices strongly correlates with the disrupted membrane anchoring mechanism. In contrast, the absence of the C-terminal linker has no impact on membrane recruitment or curved filament formation (FtsZ^Δ-Cterm^-YFP-mts). However, we found that much higher protein concentrations were required to observe FtsZ filaments on the membrane, and that these filaments were static and did not further organize into dynamic vortices (Fig. 1, right panel).

### Mutations in the FtsZ GTPase domain have a distinct effect on FtsZ ring treadmilling

In a recent study about the GTPase activity of FtsZ, Yang *et al*. were able to show that treadmilling is controlled by GTP hydrolysis and as such involved in the spatial organization of the PG synthesis machinery^12^. Based on these results, we tried to investigate the effect of altered GTPase activity on the dynamic properties of FtsZ vortices *in vitro*. For this purpose, we introduced two previously reported sequence alterations in the GTPase domain of FtsZ into the chimeric FtsZ-YFP-mts protein (D212G and D299A). In contrast to previous publications showing/describing that defects in GTP hydrolysis affect FtsZ polymerization *in vitro*^18^, our experimental conditions (0.2–0.5 µM, 4 mM GTP, 5 mM Mg^2+^) enabled the self-assembly of all chimeric protein variants into FtsZ vortices (S2 Fig, upper panels).

**Figure 2:**
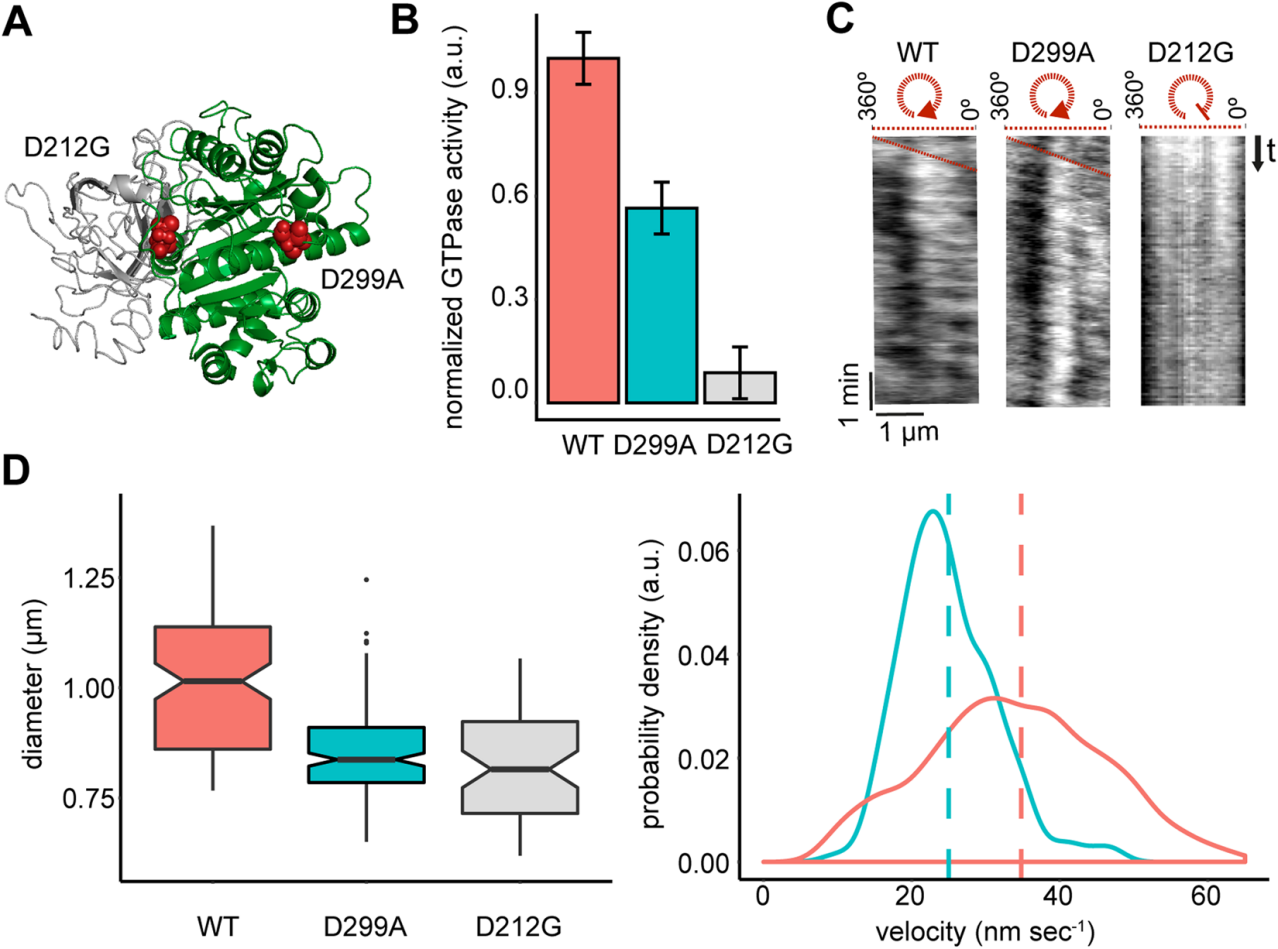
GTPase activity has a direct effect on FtsZ treadmilling. (**A**) Representative^36^ protein 3D structure (Pymol Molecular Graphics System, Version 2.3 Schrödinger, LLC^37^) highlighting (red spheres) the point mutations in the GTPase domain of FtsZ (green). (**B**) Bar plot displaying the normalized GTPase activity of each chimeric protein. Compared to the chimera with the WT moiety, [D299A] exhibits only 50% of the respective GTPase activity, while [D212G] displays an even further reduced GTPase activity. (**C**) Kymograph analysis showing a negative slope corresponding to the FtsZ treadmilling in the chimera with the WT moiety and in the [D299A] mutant. In contrast [D212G] displays no apparent slope, thus representing the static behaviour of the respective rings. These results suggest that the ring rotations of both the variant with the WT moiety and the [D299A] mutant are mediated by GTP hydrolysis (for more representative kymographs see S4 Fig, upper row). (**D**) Representative plots of the FtsZ ring diameter and velocity distributions. The colour code was kept consistent for both plots: melon - FtsZ-YFP-mts, blue - [D299A], light-grey - [D212G]. The notched boxplot in the left panel shows the median diameter of the examined FtsZ rings for each protein variant. Notches display a 95% confidence interval. Intriguingly, the mean ring diameter of the GTPase mutants seems to be smaller compared to the chimera with the WT moiety. The right panel displays the velocity distributions for the chimera with the WT moiety and the one with the [D299A] mutation. Compared to the wild-type chimera FtsZ-YFP-mts (mean ± SD = 35 ± 13 nm s^−1^, n = 1359), FtsZ^*[D299A]^-YFP-mts exhibits a reduction in the mean rotational speed (25 ± 6 nm s^−1^, n = 216) (*p* < *0.05*, t-test two-sided hypothesis). Figures were generated using R Core Team, 2019^34^, ggplot2^35^.

As shown in Fig. 2, we determined an almost tenfold reduction in FtsZ^*[D212G]^-YFP-mts GTPase activity when compared to FtsZ-YFP-mts (Fig. 2B). When considering the kymograph of FtsZ^*[D212G]^-YFP- mts, we could furthermore determine that the protein assembled into static rather than dynamic vortices of 1 µM diameter (Fig. 2C,D, S1 Movie). In contrast to previously reported *in vivo* data, FtsZ^*[D299A]^-YFP-mts did not exhibit increased GTPase activity, but rather a 2-fold decrease (Fig. 2B) when compared to the chimera with a wild type FtsZ moiety^19^. In terms of circumferential velocity, FtsZ^*[D299A]^-YFP-mts vortices showed a reduced rotational speed of 25 nm s^−1^ compared to 35 nm s^−1^ for FtsZ-YFP-mts (Fig. 2D, right panel, S2 Movie).

In summary, we conclude that our results are in line with the *in vivo* observations made by Yang *et al*. and highlight the role of the GTPase activity for FtsZ treadmilling and the resulting dynamic of the FtsZ vortices^12^.

### The membrane-targeting sequence has a direct impact on FtsZ treadmilling

**Figure 3:**
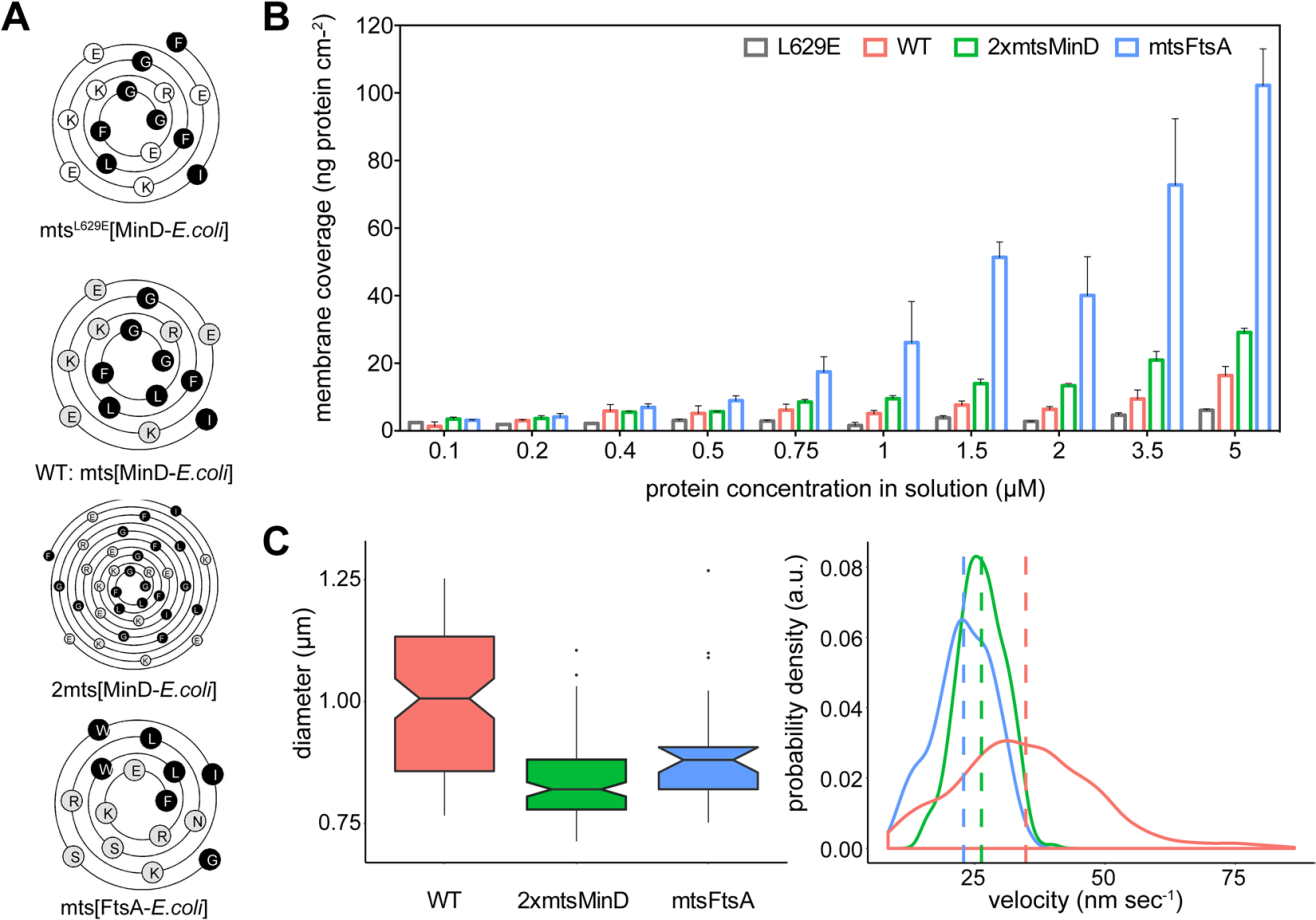
Increased membrane binding correlates with decreased treadmilling of FtsZ filaments. (**A**) Wenxiang diagrams^38^ representing the amphipathic membrane targeting sequences examined in this study. The plots depict the conical projection of each alpha-helix onto a plane, with the C-terminus of the helix in the centre and the N-terminus at the edge. Hydrophobic sides are highlighted black and hydrophilic sides in grey. (**B**) Membrane coverage in ng protein/cm^2^ of four FtsZ-YFP-mts constructs: mts [MinD-*E. coli*] (red); 2xmts [MinD-*E. coli*] (green); mts [FtsA-*E. coli*] (blue) and mts^*[L629E]^ (grey), determined via quartz crystal microbalance analysis. Error lines represent SEM of three independent experiments. Figure generated using Graphpad Prism 8.3.1^39^. (**C**) Diameter and velocity distributions of FtsZ chimera vortices. The notched boxplot in the upper panel displays the median diameter of the rings obtained for each protein variant. Notches display a 95% confidence interval. The lower panel displays the velocity shift for 2xmts [MinD-*E. coli*] (26 ± 4 nm s^−1^, n = 305) and mts [FtsA-*E. coli*] (23 ± 6 nm s^−1^, n = 228) compared to the mts [MinD-*E. coli*] displaying a mean rotational speed of 35 ± 13 nm s^−1^ (n = 1359) (*p* < *0.05*, t-test two-sided hypothesis). Representative kymographs can be seen in S4 Fig, lower row. Figures were generated using R Core Team, 2019^34^, ggplot2^35^.

To elucidate the effect of membrane tethering on the dynamic behaviour of FtsZ filaments, we generated two new FtsZ-YFP-mts variants with distinct membrane targeting sequences. Our first approach to increase FtsZ-YFP-mts membrane binding was by placement of a binary *E.coli* MinD MTS on the C-terminus of our construct (2xmts [MinD-*E. coli*], Fig. 3A). For the second variant, we used the MTS of *E. coli* FtsA (mts [FtsA-*E. coli*], Fig. 3A), one of FtsZ’s natural anchors to the membrane^20^.

As performed for the previous reconstitution experiments, the self-organization of the new chimeric variants was promoted at a protein concentration of 0.5 µM, in the presence of GTP and Mg^2+^. Both constructs were able to bind to the supported lipid bilayer system, assembled into filaments, and organized into higher-order structures (vortices) (S2 Fig, bottom panels, S3 Movie and S4 Movie).

To examine the differential binding of our mts-constructs to supported lipid bilayers, we made use of a label-free *in vitro* method: quartz crystal microbalance with dissipation monitoring (QCM-D). QCM-D allows the monitoring of the real-time attachment of proteins to artificial membrane systems through a change in the sensors resonance frequency (Δf) upon mass attachment. In our experimental approach we thus considered two different conditions for FtsZ: (i) in the presence of 4 mM GTP and Mg^2+^, conditions at which mostly long oligomers and polymers are present; (ii) in the presence of 4 mM GDP and Mg^2+^, under which short oligomers are predominant^15^. Since we were interested in the direct effect of the binding, rather than the impact of polymerization, we focused our observations on GDP related frequency changes. For this purpose, we calculated the total membrane coverage as a proxy for the membrane binding at varying protein concentrations. As displayed in Fig. 3B, affinity related changes in protein adsorption become evident in a concentration range between 0.75 to 5 μM of FtsZ (Fig. 3B). The difference in membrane coverage and thus in membrane binding is especially prominent at a concentration of 5 μM, between the control chimeric protein FtsZ-YFP-mts[L268E] (mean ± SEM = 6.1 ± 0.45 ng/cm^2^) and FtsZ-YFP-mts[FtsA-*E. coli*] (102.2 ± 10.82 ng/cm^2^) with a 17-fold increase in protein coverage. However, also compared to FtsZ-YFP-mts[MinD-*E. coli*] (16.4 ± 2.66 ng/cm^2^) and FtsZ-YFP-2xmts[MinD-*E. coli*] (29.1 ± 1.20 ng/cm^2^), FtsZ-YFP-mts[FtsA-*E. coli*] displays a significant increase in surface-attached protein density of 6-fold or 3.5-fold, respectively. Strikingly, increased membrane coverage correlates with a reduction of the rotational speed of FtsZ-YFP-mts[FtsA-*E. coli*] vortices (23 nm s^−1^, n = 228) compared to rings formed by both MinD mts chimera constructs (FtsZ-YFP-2xmts[MinD-*E. coli*]: 26 nm s^−1^, n = 305 and FtsZ-YFP-mts[MinD-*E*. coli]: 35 ± 13 nm s^−1^, n = 1359) (Fig. 3C).

### FtsZ chimeric proteins can mimic the FtsA- and ZipA-mediated membrane tethering process

Finally, we mimicked the membrane tethering process of FtsZ with its second native anchor: ZipA. For these experiments, we generated a new variant of FtsZ-YFP-mts [FtsA-*E. coli*], containing a His_6_ on the C-terminus of the chimera protein. This additional moiety allows the simulation of a high-affinity membrane tethering process, in the presence of NTA-lipids on a supported bilayer system^21,22^.

**Figure 4:**
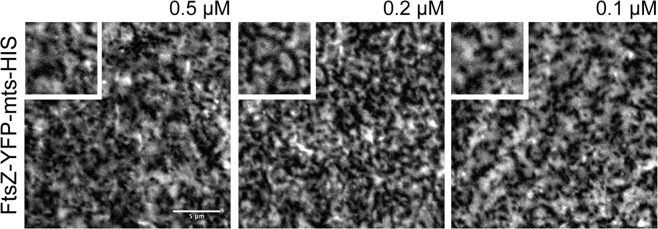
*In vitro* reconstitution of FtsZ membrane tethering mediated by ZipA, using an FtsZ-YFP-mts-His_6_-tag chimera protein. Representative snapshots of varying concentrations of FtsZ-YFP-mts-His_6_-tag, reconstituted on (*E. coli* polar lipids) supported membranes doped with 1% DGS-NTAP, in the presence of 4 mM GTP and 5 mM Mg^2+^. Small panel upper left, zoom in from each snapshot.

As previously reported by Krupka *et al*., FtsZ-sZipA (His_6_-tagged) can form circular structures in the presence of 1% DGS-NTA^23^. We thus tried to mimic the reported conditions with our His_6_-tagged fusion variant using different protein concentrations (0.5, 0.2, and 0.1 µM) (Fig. 4). As shown in Fig. 4, at early stages we observed the formation of short curved filaments and closed circular structures on the membrane, that latter were no longer visible due to bleaching effects (S5 Movie). Intriguingly, low protein concentrations correlated with the emergence of circular FtsZ structures (Fig. 4 right panel). However, these rings displayed no dynamic treadmilling but remained static. One potential reason for this defect might be the high percentage of immobile FtsZ protein on the membrane, rather than the more native situation of a static (e.g., ZipA) and exchanging (e.g., FtsZ) FtsZ protein fraction^23^.

We thus conclude that the presence of a large fraction of tightly bound filament subunits can directly inhibit and control the treadmilling of Z-rings.

## Discussion

With this work, we advance our understanding of treadmilling dynamics of one of the central components of the *Escherichia coli* divisome, FtsZ. More specifically, we provide new evidence on the biochemical modulation of FtsZ vortices and the crucial role of the membrane tethering strength for ring dynamics.

The most remarkable outcome of this study is the qualitative dependence on membrane anchoring of FtsZ treadmilling dynamics. It is known that FtsZ requires both FtsA and ZipA for membrane attachment and proto-ring formation^20^. So far, *in vitro* studies have mainly investigated the roles of either of these components. For example, Mateos-Gil *et al*.^24^ showed that ZipA-tethered FtsZ was able to form a two-dimensional network of dynamic curved filaments. Loose and Mitchison^14^ were able to reconstitute both FtsA and ZipA and showed that while FtsA-FtsZ was able to self-organize into dynamic rings, ZipA-FtsZ formed a static mesh of curved filaments, thus indicating a damping effect of ZipA on FtsZ dynamics. In our study, we also show that the presence of a static component (FtsZ-YFP-mts-His_6_-tag) leads to an immobile mesh of curved filaments. Moreover, we found a striking negative correlation of membrane binding with treadmilling speed as a result of changing the MTS on our chimera proteins. This discovery renders it tempting to speculate that the *in vivo* existence of two alternative tether proteins with different mechanisms of membrane attachment for FtsZ could be a means for controlling FtsZ treadmilling. However, natural FtsZ anchors (FtsA, ZipA) are present at different stoichiometries and may thus have a reduced effect on FtsZ dynamics when compared to the herein used membrane-targeted variant of FtsZ^14^.

We also showed that changes in GTP-hydrolysis do not affect ring formation but rather treadmilling dynamics, and that a reduction to marginal activity levels, as found for D212G that has been shown to hydrolyse GTP to less than 1% compared to the WT FtsZ^25^, is enough to abolish treadmilling. Nevertheless, we could confirm the obvious role of GTPase activity for FtsZ-ring dynamics^12,13^.

**Figure 5:**
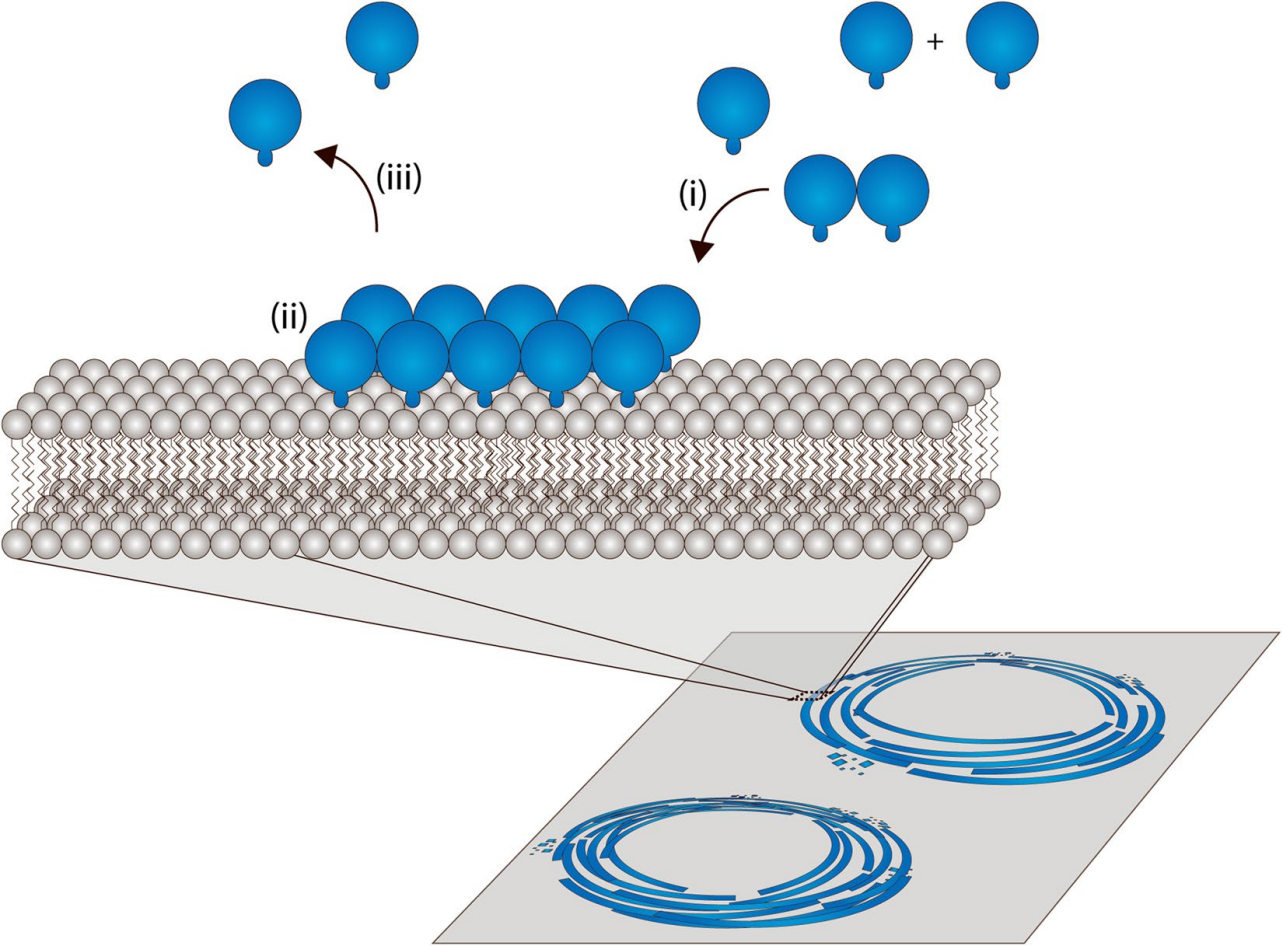
FtsZ ring dynamics depends on three factors: membrane binding, lateral interactions and GTPase activity. GTPase activity^12^ and strength of membrane binding will influence the ON (i) and OFF (iii) rates of FtsZ monomers or protofilament on the membrane. Once attached to the lipid interface (ii), lateral interactions will influence ring formation. The interplay of these three factors will determine the treadmilling speed of FtsZ-rings.

When considering the factors described above: membrane tethering and GTPase activity^12^, one can propose the following mechanism (Fig. 5). FtsZ protofilaments (pfs) will form in solution and (or) close to the membrane interface. Depending on the GTPase activity of each monomer, the filament size will be regulated (Fig. 5-i). Up to a certain threshold of FtsZ monomers (Fig. 5-i) pfs will be recruited to the membrane, and the strength of membrane binding will play a decisive role for the self-assembly into rotating rings. However, the absence of the C-terminal linker will lead to filamentous structures on the membrane, probably due to a loss in tethering flexibility^26^. Once on the membrane, specific domains in the C-terminal linker (Fig. 5-ii) will ensure the correct formation of FtsZ rings, not only keeping the right distance between the membrane and FtsZ filaments, but also between the filaments themselves^6^. Given that this particular domain properties are organism specific, further studies as performed by Sundararajan *et al*. 2017, 2018^4,27^ will be required for a comprehensive picture. Our results indicate that elimination of the CTL increases lateral interactions that affect treadmilling. In comparison a recent study by Caldas *et al*. 2020^28^ showed that ZapA/ZapB- assisted lateral interactions do not alter treadmilling. The most plausible explanation is the distance between protofilaments in the polymer cluster (overlapping polymer length)^29^. ZapA/B are proteins that crosslink the protofilament, thus increasing the distance parameter between filaments and the polymer dynamics, when compared with the bundles in this study^2^. In addition, ZapA-FtsZ interactions are weak and transient leading in almost no effect on treadmilling. Lastly, treadmilling will be dictated by (Fig. 5-ii) membrane binding and GTP-hydrolysis. Intriguingly, we observed that the reduction of GTP-hydrolysis and the increase of membrane binding resulted in a delay of filament breakage and a subsequent decrease of FtsZ treadmilling speed. We propose that these two factors influence the rate of rupture by altering the on- (Fig. 5-i) and off-rates (Fig. 5-iii) of membrane association. In the presence of static components, like ZipA (or ZipA-mimics), the rotational speed of the septal ring will also be affected. We propose that *in vivo*, a balance between FtsA and ZipA can dictate the rate of treadmilling and therefore impact the cell-division rate.

In summary, our *in vitro* study provides new insights about Z-ring architecture and identifies key factors that regulate the formation and dynamic of FtsZ vortices both *in vitro* and *in vivo*^12,13^. In the future, we propose to further consider additional proteins of the division ring or even peptidoglycan synthesis components to examine their effect on FtsZ treadmilling dynamics. Moreover, we want to highlight the use of the generated chimera proteins (FtsZ-YFP-mts-His_6_ variant and FtsZ-YFP-mts [FtsA-*E. coli*]) as possible components for a minimal synthetic cell-division machinery, as they allow a further reduction on the number of elements previously used in reconstitution experiments^14,23^.

## Materials and Methods

### Cloning

FtsZ-YFP-mts variants were constructed through site-directed mutagenesis or whole sequence replacement or displacement using the oligonucleotides specified in (S1 Table). In the case of the FtsZ^Δ-Cterm^-YFP-mts chimera, we removed amino acids 315–383 (*E. coli* FtsZ sequence). After Dpn I digestion, vector constructs were transformed into competent *E.coli* CH3-Blue cells and cultured in LB medium containing 50 g/ml ampicillin (37 °C, ON). DNA purification was performed according to the manufacturer’s instructions (NucleoBond Xtra Midi, Macherey-Nagel, Düren, Germany). Successful plasmid construction was confirmed through next-generation sequencing (Eurofins, Brussel, Luxembourg).

### Protein purification

All proteins were purified as previously described^16^. Briefly, the proteins of interest were cloned into pET-11b expression vectors or pET-28a. Expression vectors were transformed into *E. coli* BL21 DE3 and grow at 37 °C ON in the presence of ampicillin. For protein over-expression, LB medium was inoculated and cells were grown to an OD of 0.8 until protein synthesis was induced through 0.5 mM IPTG. Then, cells were incubated at 20 °C ON. Cells were sonicated and centrifugation at 3200 rpm (4 °C). Subsequently, proteins of interest were precipitated from the supernatant through 30% ammonium sulphate and a 20 min incubation on ice (slow shaking). After centrifugation (3200 rpm, 4 °C) and re-suspension of the pellet, proteins were purified by anion exchange chromatography using a 5 × 5 ml Hi-Trap Q-Sepharose column (GE Healthcare, Chicago, USA). The purity of the proteins was confirmed by SDS-PAGE and mass spectrometry.

### GTPase activity assay

FtsZ GTPase activity was determined utilizing the BIOMOL GREEN reagent for colorimetric phosphate quantification (Enzo Life Sciences, Lörrach, Germany). For this purpose, 1 mM GTP was added to a 5 µM stock of FtsZ in reaction buffer (50 mM Tris pH 7.5, 300 mM KCl, 5 mM MgCl_2_). Subsequently, FtsZ-GTP (each 13 µL) was added to a well plate containing 37 µL stop solution (70 mM Tris pH 7.5, 200 mM KCl, 65 mM EDTA). This step was repeated for a total of 7 points each spaced by 20 second intervals (time points: 0, 20, 40, 60, 80, 100 and 120 s). For activity determination, a standard curve was prepared using a 1:2 serial dilution of 40 µM Phosphate (Pi standard) in water. Blacks for both standard and sample series were prepared containing either 50 µL water or stop solution, respectively. Then, 100 µL BIOMOL GREEN reagent were added to each well and absorption was followed at 620 nm in a Spark multimode microplate reader (Tecan Group Ltd, Männedorf, Switzerland).

### Small unilamellar vesicles (SUVs) for SLBs on glass and on quartz crystal sensors

Either DOPC:DOPC, 70:30 mol% (quartz) or *E. coli* polar lipid extract (glass; Avanti, AL, USA), initially dissolved in chloroform, were dehumidified under a gas nitrogen stream. Chloroform traces were further removed through desiccation (1 h). Subsequently, the lipid film was hydrated with SLB-buffer (50 mM Tris-HCl, 150 mM KCl, pH 7.5) to a final lipid concentration of 4 mg/ml^−1^. Then, multilamellar *E.coli* polar lipid extract vesicles were sonicated (10 min) to obtain small unilamellar vesicles (SUVs). In contrast, a freeze-thaw protocol (8 cycles) with successive extrusion (37 ×, 50 nm Whatman membrane) was used to obtain DOPC:DOPG SUVs^30^.

### Supported lipid bilayers on glass (SLBs)

# 1.5 glass coverslips (Menzel, Germany) were cleaned in air plasma for 10 min at 60 W (0.3 mbar). Then a plastic chamber was attached to the cleaned coverslip using an ultraviolet-curable glue (NOA 63, Norland Products Inc., USA). The SUV stock was diluted in SLB buffer (50 mM Tris-HCl at pH 7.5, 150 mM KCl) to 0.5 mg ml−1 and 75 μl were added to the reaction chamber. CaCl_2_ was further subsidized to a final concentration of 3 mM to promote vesicle fusion and the formation of a lipid bilayer on the glass surface. The chambers were then incubated at 38 °C for 20 min and subsequently washed with pre-warmed SLB buffer to remove non-fused vesicles. Finally, SLBs were washed with reaction buffer (50 mM Tris-HCl at pH 7.5, 150 mM KCl and MgCl2 (5 mM or 1 mM)).

### QCM-D Measurements

Quartz crystal sensors (Biolin Scientific, Gotheburg, Sweden) were pre-treated with piranha-solution (H_2_SO_4_:H_2_O_2_, 3:1; 1 h), thoroughly cleaned with ddH_2_O and dried under a nitrogen stream. Subsequently, sensors were immediately mounted in the flow cells of the QSense Analyzer (Biolin Scientific, Gotheburg, Sweden) and the crystals resonance frequencies were obtained in air and reaction buffer (QSoft Version 2.5.36; Biolin Scientific, Gotheburg, Sweden). Second signature, S2, values were calculated to check for the systems performance according to the equation indicated in Cho *et al*. (S2 Table)^30^. After the stabilization of the resonance frequency in response to the deposition of ions on the measurement surface (flow rate: 0.15 ml/min), 1 mg/mL SUVs (DOPC:DOPC, 70:30 mol %) with 5 mM CaCl_2_ were introduced to the crystal. Successful SLB formation was verified through a characteristic frequency change (Δf) of 25 ± 1 Hz^30^. Succeeding the formation of a signal base line, the flow rate was reduced to 0.1 ml/min and protein solutions between 0.1 to 5 uM, with either 4 mM GTP or GDP, were successively adsorbed to the SLB and desorbed under constant flow (0.1 ml/min). Raw data export was performed using QTools 3 Version 3.1.25.604 (Biolin Scientific, Gotheburg, Sweden) and extraction of binding event related frequency changes was executed using a customized MATLAB R2018a (The MathWorks, Inc., Natick, USA) script. Protein specific membrane coverages were calculated according to the Sauerbrey equation^31^ and data visualization was performed with GraphPad Prism 7.0d (GraphPad Software, La Jolla, USA). All measurements were performed under constant temperature (24 °C). If not indicated otherwise, all in the following used data depict frequency changes of the 9^th^ overtone.

### Self-organization assays

Either 0.5 µM or 0.2 µM FtsZ-YFP-mts or FtsZ-YFP-mts-mutants were added to the SLB coated reaction chamber (200 μl reaction volume). Polymerization was induced with 4 mM GTP.

### Total internal reflection fluorescence microscopy (TIRF)

All experiments were performed on a WF1 GE DeltaVision Elite total internal reflection fluorescence microscope (GE Healthcare Life Sciences, Germany) equipped with an OLYMPUS 100x TIRF objective (NA 1.49; Olympus K.K., Tokio, Japan) and the DeltaVision Elite system (GE Healthcare, Chicago, USA). Images were acquired with a PCO sCMOS 5.5 camera (PCO AG, Kelheim, Germany) controlled by the softWoRx Software (GE Healthcare, Chicago, USA). All introduced FtsZ-YFP-mts variants were excited at 488 nm (diode laser: 10 mW, before objective). A standard FITC filter set was used for fluorescence imaging. For time-lapse experiments, images were acquired every 3 or 10 sec, with a 0.05 sec exposure time and shut light illumination between acquisitions.

### Image analysis and processing

Image analysis was carried out using MATLAB 2015 (MATLAB and Image Processing and Computer Vision Toolbox 2015a; The MathWorks, Inc., Natick, USA^32^) and Fiji^33^. Images correspond to an average of 5–10 frames for each time-series experiment. For the kymograph analysis, time-series acquisitions were filtered using the standard mean filter and the drift correction plugin of Fiji. Subsequently, we used a customized MATLAB script which allowed for the definition of a ring as two specific coordinates and the automatic calculation of a circle with a matching radius r defined by the FtsZ ring. Then, three trajectories corresponding to three concentric circumferences (r, r + 1 and r − 1 pixels) were determined. Next, the script will read the time-series data and calculate a kymograph for each time point and trajectory. The final kymograph thus corresponds to the average of the three different trajectories. At this point the user will have to manually identify a line of the kymograph and from here the slope will be calculated. On a next step the algorithm will also automatically calculate the slope. For this step, we first smoothed the kymograph with a 2^nd^ order Savitzky-Golay filter and enhanced the contrast using a contrast-limited-adaptive-histogram-equalization (CLAHE) routine (MATLAB). To then identify characteristic pattern frequencies on the kymograph, Fourier analysis was performed and the depicted slopes correspond to the phase changes at the specific frequency. It will automatically analyse vertical regions of 50 pixels and find the vertical frequency using Fourier analysis. A periodicity of vertical patterns will be found and the signal will have a specific phase and frequency. By calculating the phase shift at a given circulation frequency, we can extract the slope in the kymograph. Given that the algorithm sweeps the kymograph, we will have a distribution of slopes and the final output will be the mean of this distribution. Quality criteria were chosen to reject low quality regions over the kymograph.

The slope (manual or automatic) will be used to calculate the time-dependent treadmilling velocity for each ring under the assumption that FtsZ rings rotate in a directional fashion.

We cross-validated the automatically calculated slope with the manually calculated slope and found that they are statistically comparable (S5 Fig). Since the automatically calculated slope is less affected through human bias we decided to use this data for our further analysis.

### Data visualisation and statistical analysis

Data visualisation and statistical tests were performed in R (R Core Team, 2019^34^) and Figs. 2B,D and 3C were produced using the package ggplot2^35^. Figure 3B was generated using Prism (Graphpad Prism 8.3.1.). Boxplots display five summary statistics (media, two hinges and two whiskers) and all ‘outlying points’. The lower and upper hinges correspond to the first and third quartiles. The notches extend 1.58 * IQR / sqrt(n), thus providing a 95% confidence interval for the comparison of medians values. A two-sided t-test was used to analyse the statistical difference between the WT treadmilling mean and the generated mutant variants.

## Supplementary information

Supplementary Information.

SSupplementary Movie S5.

Supplementary Movie S4.

Supplementary Movie S3.

Supplementary Movie S1.

Supplementary Movie S2.
