# Supplementary material for: The speed of FtsZ treadmilling is tightly regulated by membrane binding
Source: Sci Rep. 2020 Jun 26;10:10447. doi: 10.1038/s41598-020-67224-x (PMC7320174; doi:10.1038/s41598-020-67224-x)
Supplement: Supplementary file 1 — Supplementary Information. [file 41598_2020_67224_MOESM1_ESM.docx]

**The speed of FtsZ treadmilling is tightly regulated by membrane binding**

Daniela A. García-Soriano^1,2,4^, Tamara Heermann^1^, Ana Raso^1,3^, Germán Rivas^3^ and Petra Schwille^#1^

**S1 Table**. Primers used to generate FtsZ-YFP-mts variants.

| **Primer name** | **Sequence (5’ – 3’)** |
| --- | --- |
| FtsZ-YFP-mts*[L629E]_FW | atcctccgaacTCgcgtttgagg |
| FtsZ-YFP-mts*[L629E]_RV | cctcaaacgcGAgttcggaggat |
| FtsZ*[Δ-CTL]-YFP-mts_FW | acctgtcgcaacaacggtta |
| FtsZ*[Δ-CTL]-YFP-mts_FW | taaggatccggctgctaacaaag |
| FtsZ*[D299A]-YFP-mts_FW | TCATATCCGGGGCAAGAGAAGTAC |
| FtsZ*[D299A]-YFP-mts_RV | GTACTTCTCTTGCCCCGGATATGA |
| FtsZ*[D212G]-YFP-mts_FW | acggtgcgtacgcctgcaaagtcca |
| FtsZ*[D212G]-YFP-mts_RV | tggactttgcaggcgtacgcaccgt |
| FtsZ*[T108A]-YFP-mts_FW | GGTGGTGGTgccGGTACAGGT |
| FtsZ*[T108A]-YFP-mts_RV | ACCTGTACCggcACCACCACC |
| FtsZ-YFP-2mts_FW | caaacgcttgttcggagg |
| FtsZ-YFP-2mts_RV | cctccgaacaagcgtt |
| FtsZ-YFP-mtsFtsA_FW | ATTGAGTCGCTTGATCCACGAGCCgcggccgcccttgta |
| FtsZ-YFP-mtsFtsA_RV | AGTTGGCTGCGAAAAGAGTTTTAAggatccggctgctaacaaag |

**S2 Table**. S2 signature values for the analysis of FtsZ-YFP-mts membrane binding affinities examined with the Quartz crystal microbalance with dissipation monitoring (QCM-D). Reference values for harmonics 3, 5, 7, 9, 11 and 13 were obtained from Cho et al.: 2.45, 2.5, 2.49, 2.45, 2.46 and 2.47, respectively.

|  |  | **GTP** | | | **GDP** | | |
| --- | --- | --- | --- | --- | --- | --- | --- |
|  | **Overtone** | **replicate 1** | **replicate 2** | **replicate 3** | **replicate 1** | **replicate 2** | **replicate 3** |
| **mts[FtsA-E.coli]** | 1 | 2,37 | NA | 2,32 | 2,28 | 2,33 | 2,28 |
|  | 3 | 2,48 | NA | 2,45 | 2,45 | 2,43 | 2,47 |
|  | 5 | 2,45 | NA | 2,39 | 2,43 | 2,39 | 2,52 |
|  | 7 | 2,52 | NA | 2,47 | 2,45 | 2,43 | 2,50 |
|  | 9 | 2,26 | NA | 2,46 | 2,45 | 2,40 | 2,49 |
|  | 11 | 2,56 | NA | 2,46 | 2,49 | 2,54 | 2,52 |
|  | 13 | 2,55 | NA | 2,45 | 2,47 | 2,65 | 2,52 |
| **mts[MinD-E.coli]** | 1 | 2,28 | 2,28 | 2,34 | 2,27 | 2,18 | 2,17 |
|  | 3 | 2,43 | 2,46 | 2,45 | 2,45 | 2,42 | 2,45 |
|  | 5 | 2,41 | 2,43 | 2,41 | 2,42 | 2,47 | 2,46 |
|  | 7 | 2,47 | 2,47 | 2,43 | 2,45 | 2,45 | 2,48 |
|  | 9 | 2,46 | 2,43 | 2,39 | 2,45 | 2,45 | 2,45 |
|  | 11 | 2,48 | 2,44 | 2,45 | 2,62 | 2,48 | 2,48 |
|  | 13 | 2,47 | 2,41 | 3,34 | 2,67 | 2,48 | 2,52 |
| **2mts[MinD-E.coli]** | 1 | 2,18 | NA | 2,36 | 2,17 | 2,21 | 2,40 |
|  | 3 | 2,43 | NA | 2,41 | 2,43 | 2,46 | 2,48 |
|  | 5 | 2,42 | NA | 2,42 | 2,39 | 2,48 | 2,46 |
|  | 7 | 2,49 | NA | 2,44 | 2,44 | 2,52 | 2,47 |
|  | 9 | 2,47 | NA | 2,35 | 2,44 | 2,52 | 2,51 |
|  | 11 | 2,48 | NA | 2,52 | 2,47 | 2,69 | 2,52 |
|  | 13 | 2,45 | NA | 1,78 | 2,45 | 2,64 | 1,36 |
| **mts*[L629E]** | 1 | 2,26 | 2,34 | 2,19 | 2,21 | 2,20 | 2,21 |
|  | 3 | 2,45 | 2,47 | 2,45 | 2,44 | 2,44 | 2,42 |
|  | 5 | 2,43 | 2,46 | 2,39 | 2,42 | 2,41 | 2,39 |
|  | 7 | 2,46 | 2,49 | 2,46 | 2,48 | 2,48 | 2,46 |
|  | 9 | 2,44 | 1,05 | 2,41 | 2,47 | 2,49 | 2,42 |
|  | 11 | 2,45 | 2,51 | 2,41 | 2,61 | 2,51 | 2,47 |
|  | 13 | 2,39 | 2,52 | 2,41 | 2,73 | 2,47 | 2,53 |


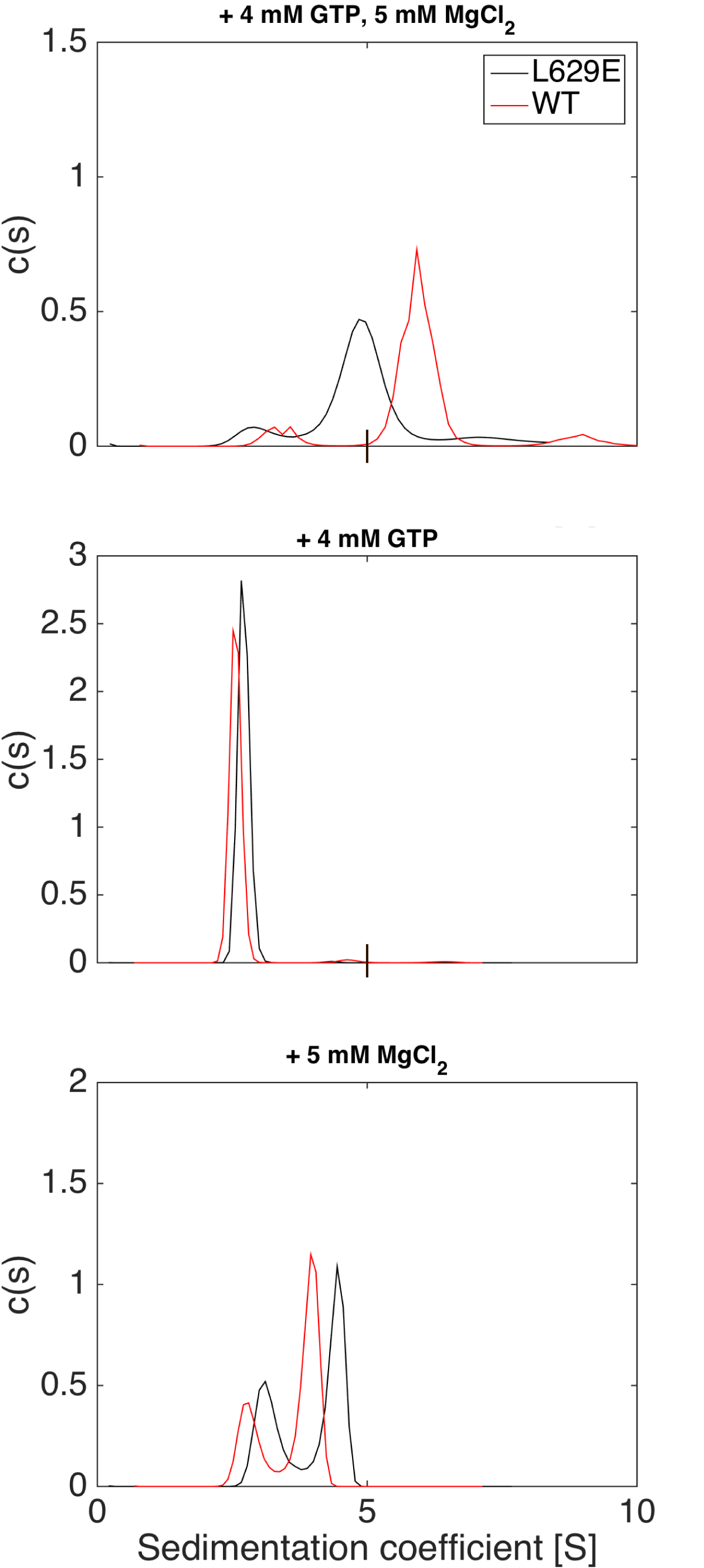


**S1 Fig.** Distribution of sedimentation coefficients obtained from sedimentation velocity analysis of FtsZ-YFP-mts (WT) and FtsZ-YFP-mts*[L629E]. Upper panel: In the presence of both GTP and Mg^2+^ the sedimentation-velocity profile shows that both proteins are polydisperse, indicating the presence of polymers of various size. Middle panel: Only when GTP is present, a main peak is observed in the sample, which is comparable to the monomeric protein. Lower panel: When Mg^2+^ is present, we observed a main peak (oligomers) as well as the presence of monomers (second peak with a decreased sedimentation coefficient).


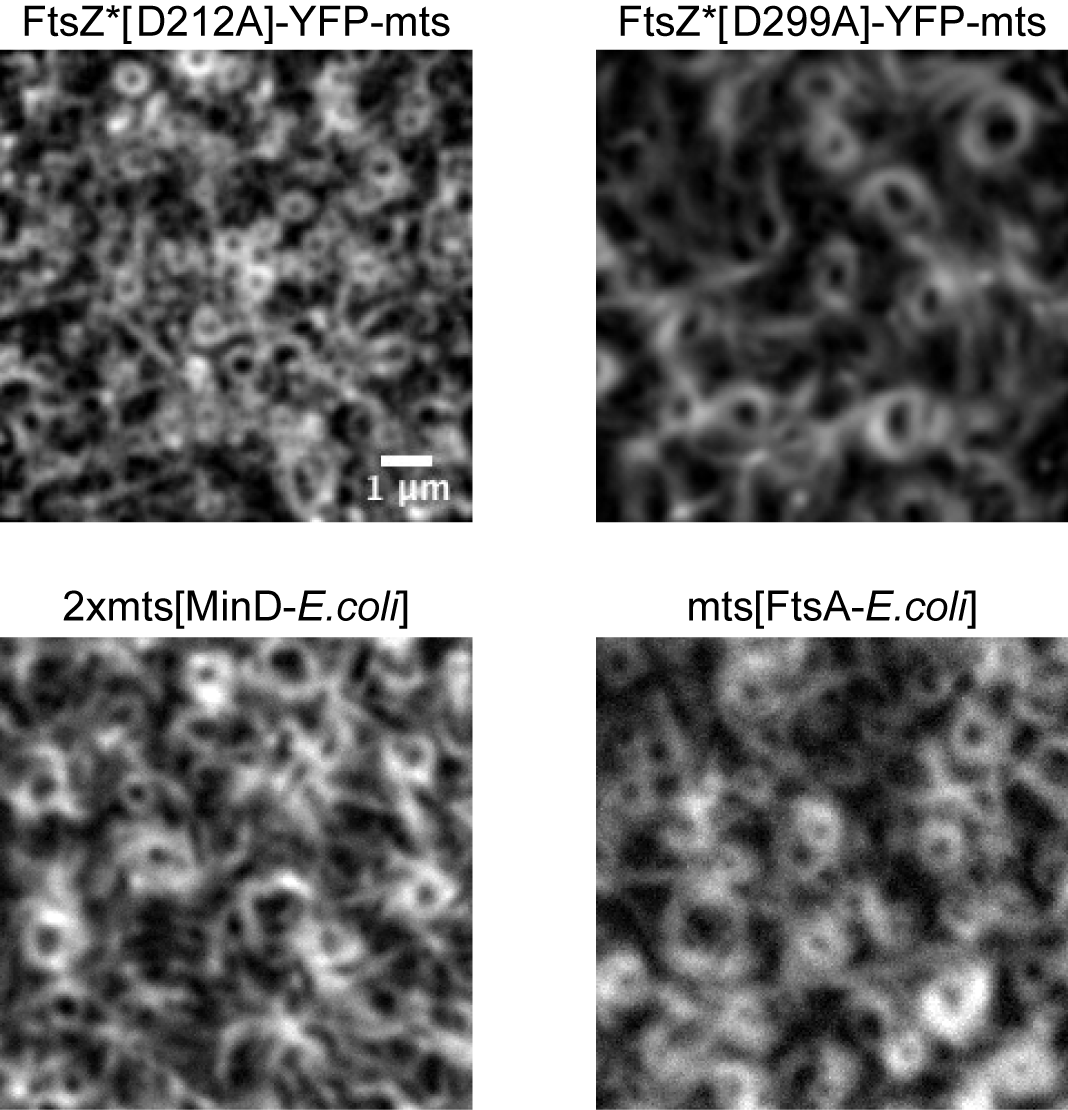


**S2 Fig.** Representative snapshots of the *in vitro* reconstitution of FtsZ*[D212A]-YFP-mts (upper left panel), FtsZ*[D299A]-YFP-mts (upper right panel), 2xmts[MinD-*E. coli*] (lower left panel) and mts[FtsA-*E. coli*] (lower right panel). Scale bar indicates 1 μm.


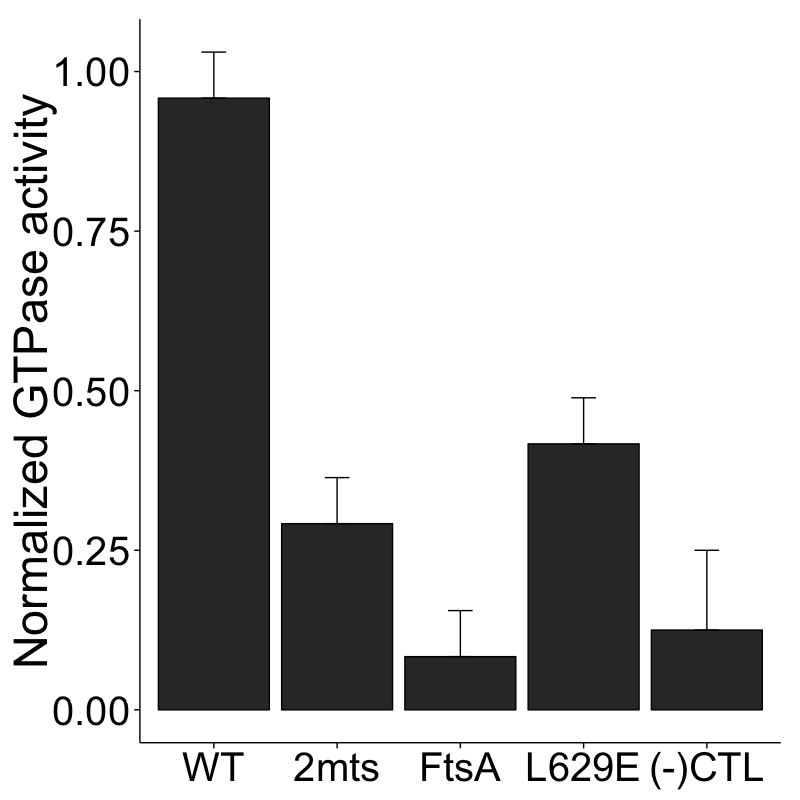


**S3 Fig.** Normalized GTPase activity of FtsZ-YFP-mts (WT) compared to 2xmts [MinD-E. coli], mts[FtsA-E. coli], FtsZ-YFP-mts*[L629E] and FtsZΔ-Cterm-YFP-mts. All examined protein variants display a reduced GTPase activity, when compared to the chimera with the WT moiety.


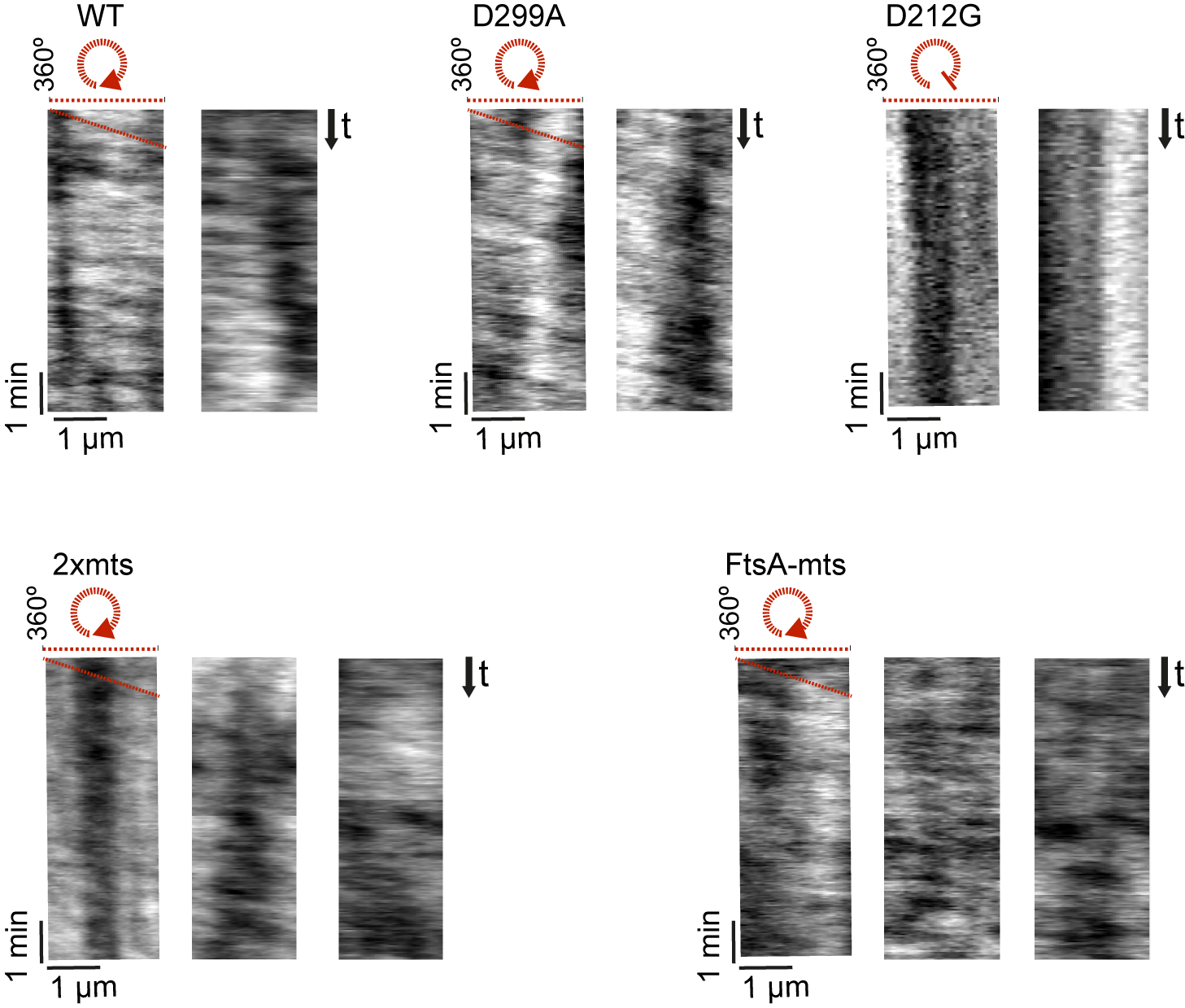


**S4 Fig.** Representative kymographs for each chimera protein used in this study. Upper row: FtsZ-YFP-mts (WT), FtsZ^*[D299A]^-YFP- mts (D299A) and FtsZ^*[D212G]^-YFP- mts (D212G). Lower row: FtsZ-YFP-2xmts [MinD-E. coli] (2mts), FtsZ-YFP-mts[FtsA-E. coli] (FtsA-mts).


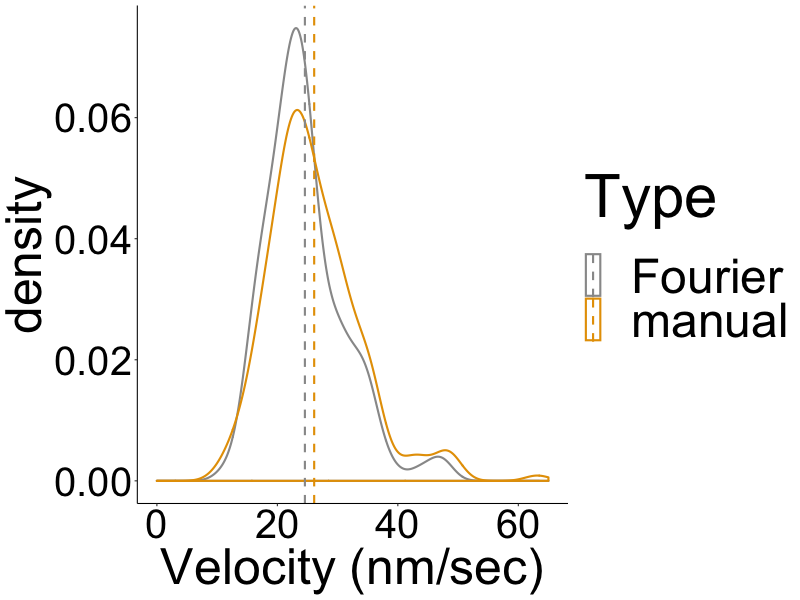


**S5 Fig.** Validation of Fourier slope prediction (gray) against manually calculated slope (yellow) using a subset from the data obtained after analyzing the D299A samples. Manual calculated slope (mean ± SD = 26 ± 8 nm s^-1^, n = 288), Fourier slope prediction (mean ± SD = 26=5 ± 7 nm s^-1^, n = 114), (*p > 0.05*, t-test two-sided hypothesis). The values are statistically similar and validate the Fourier slope prediction data.

**S1 Movie.** FtsZ^*[D212G]^-YFP- mts assembles into static rings. 101 frames, taken every 3 sec.

**S2 Movie.** FtsZ^*[D299A]^-YFP- mts assembles into dynamic vortexes. 101 frames, taken every 3 sec.

**S3 Movie.** FtsZ-YFP-2xmts [MinD-E. coli] assembles into dynamic vortexes. 101 frames, taken every 3 sec.

**S4 Movie.** FtsZ-YFP-mts[FtsA-E. coli] assembles into dynamic vortexes. 101 frames, taken every 3 sec.

**S5 Movie.** FtsZ-YFP-mts-His_6_-tag assemble into static rings. 21 frames, taken every 3 sec.
